# Contribution of neuroimaging in late-life depression

**DOI:** 10.1192/j.eurpsy.2023.56

**Published:** 2023-07-19

**Authors:** T. Desmidt

**Affiliations:** ^1^CHU de Tours; ^2^UMR 1253, iBrain, Université de Tours, Inserm; ^3^CIC 1415, CHU de Tours, Inserm, Tours, France

## Abstract

**Abstract:**

Late-life depression (LLD) is currently a hot topic for neuroimaging studies, while an increasing number of imaging modalities are now available for the characterization of brain structure and function. Changes in brain volumes, including hippocampal volume, have been observed in LLD, although results are inconsistent. Vascular lesions are more consistently associated with LLD and may constitute factors of poor prognosis. More recent MRI modalities, including fMRI and DTI, also show interesting results, notably to assess treatment response in LLD. Molecular Imaging also has the potential to improve our understanding of the pathophysiology of LLD, using imaging such as PET-FDG and Amyloid PET. Finally, there are emerging studies with novel neuroimaging modalities such as Ultrasound to measure subtle mechanical properties in the brain of patients with LLD. Finally, we contend that neuroimaging has the potential to provide markers for the identification of subcategories of LLD (vascular depression, amyloid depression, etc.) as well as prognostic values and markers for treatment response.

**Disclosure of Interest:**

None Declared

